# Current Advances and Hurdles in Chimeric Antigen Receptor Technology

**DOI:** 10.3390/cancers12113329

**Published:** 2020-11-11

**Authors:** Scott McComb, Seung-Hwan Lee

**Affiliations:** 1 Human Health Therapeutics Research Centre, National Research Council Canada, Ottawa, ON K1A 0R6, Canada; 2 Department of Biochemistry, Microbiology, and Immunology, Faculty of Medicine, University of Ottawa, Ottawa, ON K1H 8M5, Canada; 3 The University of Ottawa Centre for Infection, Immunity, and Inflammation, Ottawa, ON K1H 8M5, Canada

Since tumor-specific T cells were first utilized to treat melanoma patients in 1986 [1], immune cell-based therapy for cancer treatment has been a topic of continuous immunological research and development, despite ups and downs in the broader interest in the subject. One popular form of cancer immunotherapy is chimeric antigen receptor (CAR) technology, wherein an extracellular antigen-binding domain, usually composed of an antibody-derived single-chain variable fragment (scFv) domain, confers antigen-specific receptor activity [2]. This extracellular targeting domain is synthetically recombined with intracellular signaling domains, which can be derived from many immune cell signaling molecules [3]. CARs can then be delivered into immune cells (most commonly Natural Killer (NK) or T cells) using viral vectors or other gene delivery techniques, resulting in a living drug that can seek and destroy cancer cells. Following sensational results in clinical trials over the last decade, the United States Food and Drug Administration (FDA) has so far approved three CD19-targeted CAR therapies for patients with B-cell hematological malignancies, such as lymphoma and leukemia [4]. This work has been followed more recently by many ongoing trials using various molecular strategies to target multiple antigens for the treatment of B-cell family cancers. There is also an increasing number of ongoing CAR-T clinical trials for other types of blood cancer and solid tumors, suggesting that more innovative CAR therapies will soon be available to patients who have had no other traditional options of treatment [3]. This Special Issue is dedicated to highlighting current advances in chimeric antigen receptor technology and invites manuscripts that report strategies to overcome existing obstacles to wider development and clinical adoption of CAR therapies. Those include, but are not limited to, the challenges described below.

## Improving Specific Recognition of Tumor Cells

The extracellular domains that specifically recognize tumor antigens are critical for creating a functional CAR which can specifically target cancer cells with minimal on- or off-target toxicity. The scFv, consisting of the light-chain and heavy-chain variable domains connected by a flexible linker, is still the most common antigen binding domain used in CAR designs [4], but other formats such as camelid single-domain antibodies (sdAb; also called nanobodies) are now making their way into the clinic [5]. Despite the great number of papers now being published in the CAR field of research, the process of selecting an antigen-binding domain for CAR application remains a frustratingly empirical process. Simple but important questions such as how antibody affinity relates to CAR function remain largely unanswered [6]. Papers addressing the relationship between the immunochemical properties of antibodies and CAR signaling would be of significant interest. 

## Potent CAR Signaling

Throughout multiple generations, CAR constructs have evolved to implement various intracellular signaling domains for stronger signaling upon the ligand binding. Sequentially, CD3ζ was first added, followed by costimulatory domains, and recently cytokine payload or cytokine receptor domains are also being incorporated to improve the potency of CAR signaling [3]. The inclusion of such cytokine payloads may be an important component to unlocking the potential of NK cells as a source for CAR cell therapy [7]. In addition, the optimization of the hinge and transmembrane region is required for better formation of the immunologic synapse. 

## CAR Toxicity

Even though the CAR therapies show dramatic efficacy for relapsed B-cell family cancers, cytokine release syndrome (CRS) and neurotoxicity remain significant medical challenges with these therapies [8,9]. CRS occurs when CAR T cells become potently activated by target cells during the acute phase of response, leading to overwhelming activation and the release of cytokines, such as IL-6, IFN-γ, and TNF-α. Even though tocilizumab, an anti-IL-6 receptor antibody, has been found to be a potent treatment for CRS [9], alternate strategies to dampen or otherwise control the hyperactivation of CAR T cells and the associated macrophage activation syndrome would be beneficial to CAR-T clinical therapy. Acute neurologic toxicity is another important complication associated with CAR-T therapy, where therapeutic interventions are not yet as well understood as for CRS. The cause of neurotoxicity is still not clear, although intriguing new work suggests that expression of CD19 in certain brain cells may play a role [10]. The development of in vitro and in vivo models to investigate the incidence of neurologic or CRS toxicity is urgently needed.

## Allogeneic CAR Therapy

The extreme economic burden of CAR-T therapy is a critical barrier to expanding its use to more common and more deadly cancer types. Strategies to develop a potentially inexhaustible and much less costly supply of “off-the-shelf” allogeneic CAR-T therapies are now making rapid progress [11,12]. Genome editing strategies based on CRISPR-Cas9 or other technology can now be used quite effectively at the laboratory level to reprogram the properties of T cells at the laboratory scale and is now breaking through into clinical application in the last year [12]. Studies that provide deeper insights into how to better design, develop, and deploy gene-edited CAR therapies would be of high value to the field. 

## CAR-NK Therapy

NK cells are another cell type that has been used as an off-the-shelf CAR treatment [13]. The scalability of such CAR-NK therapies could potentially reduce the cost, toxicity, and complexity of genetically modified cell therapies. Furthermore, CAR-NK cells express multiple tumor-specific activating receptors, minimizing the likelihood of tumor cell escape through the downregulation of the CAR target antigens. However, NK cells are more resistant to genetic manipulation, hindering CAR applications. Additionally, infused NK cells have shown limited persistence, requiring multiple infusions and supplemental IL-2/15 injections. Other strategies, including rapid NK cell expansion in vivo, different sources of NK cells such as pluripotent stem cells, and CAR constructs with NK cell-specific signaling domains, are being incorporated for robust immunotherapy. 

## Relapse

The novelty of CAR-T therapy means that most patients participating in CAR T-cell trials have only been followed for a relatively short time, and particularly for solid tumor-targeting CARs, we still lack enough data to evaluate cancer relapse for extended periods of CAR therapy in humans. It is true that, in many preclinical animal studies involving CAR treatment, relapses can be observed but are only rarely studied in detail. Relapses could be due to the tumor cells mutating or downregulating the expression of the targeted tumor antigen [14]. To avoid tumor evasion from a CAR targeting one tumor antigen, infusion of a homogeneous population of either T or NK cells expressing a bi-specific CAR construct with dual tumor antigen specificity or the infusion of a heterogeneous mixture of T and NK cells expressing CARs targeting two different tumor antigens may be effective strategies [14]. Studies investigating the development of novel strategies to counteract tumor heterogeneity or immunoevasion would be of value.

The rapid advancement in cellular immunotherapy in recent years such as CAR-T therapy is exemplified by recent FDA approval of three forms of CAR-T therapy for the treatment of B-cell malignancies. While these therapies are already resulting in durable remissions within some patients, there is a long way to go before all cancer patients have access to effective, affordable, and safe CAR therapies. Innovative ideas to overcome the challenges described above will potentiate CAR therapy as a mainstay for immunotherapy against cancer.
